# Compendium of hand, foot and mouth disease data in Malaysia from years 2010–2017

**DOI:** 10.1016/j.dib.2019.103868

**Published:** 2019-03-20

**Authors:** Bryan Raveen Nelson, Hisham Atan Edinur, Mohd Tajuddin Abdullah

**Affiliations:** a Institute of Tropical Biodiversity and Sustainable Development, Universiti Malaysia Terengganu, 21030 Kuala Nerus, Terengganu, Malaysia; b School of Health Sciences, Universiti Sains Malaysia, 16150 Kubang Kerian, Kelantan, Malaysia; c School of Marine and Environmental Sciences, Universiti Malaysia Terengganu, 21030, Kuala Nerus, Terengganu, Malaysia

## Abstract

This data article is constructed using information from hand, foot and mouth disease outbreaks in every Malaysian state. Important information like total number of cases from every state and age group, humidity, air temperature and wind as well as types of viruses isolated from patients infected with hand, foot and mouth disease were gathered and sorted into four tables. The information sources include Malaysian Administrative Modernisation and Management Planning Unit (MAMPU), National Centre for Biotechnology Information (NCBI) and National Oceanic and Atmospheric Administration (NOAA). Since the Ministry of Health Malaysia only begun archiving hand, foot and mouth disease cases between years 2010–2017 in MAMPU, weather data extractions are following the similar timeline. Conversely, published data on viruses and their serotypes associated to hand, foot and mouth disease were extracted from NCBI but, data availability were limited between the years 2010 and 2014.

**Table:** Specifications table

Subject area	*Medicine and Dentistry*
More specific subject area	*Infectious disease*
Type of data	*Table*
How data was acquired	*Data mining from web portals of Malaysian Administrative Modernisation and Management Planning Unit, National Centre for Biotechnology Information and National Oceanic and Atmospheric Administration*
Data format	*Raw*
Experimental factors	*Cases (number of infected by hand, foot and mouth disease) were arranged into age category, Malaysian states, months and years. Weather properties (temperature, humidity and wind) were arranged into monthly and yearly data. Viruses associated with hand, foot and mouth disease were arranged by serial numbers, serotypes, Malaysian states, months and years.*
Experimental features	*Reported cases of hand, foot and mouth disease, viral serotypes, weather conditions during outbreaks, published nucleotide sequences, keywords used, authenticity of data source and human error during calculations.*
Data source location	*National Oceanic and Atmospheric Administration* *-Weather stations a) Pulau Pinang (Bayan Lepas) – N 05°17′59″, E 100°15′37″* *b) Melaka (Jalan Kota Laksamana) – N 02°11′45″, E 102°14′25″* *c) Sarawak (Kuching International Airport) – N 01°29′04″, E 110°20′48″* *-Web portals**http://tgftp.nws.noaa.gov* *http://www.data.gov.my* *https://www.ncbi.nlm.nih.gov*
Data accessibility	*All data are available in this article*
Related research articles	*N.K.A. Khalib, N.J. Shafie, H.H. Basri, B.R. Nelson, M.T. Abdullah, Non-volant small mammal data from fragmented forests in Terengganu State. Data Brief 21 (2018) 1514–1520.*

**Table:** 

**Value of the data** • Outbreak raw data is tangible for disease etiology and its emergence in Southeast Asia. • Virulence data are useful for descriptive epidemiology like secular trends, seasonality, epidemic period, geography and person attributes (disease-age and disease-scale) relationships. • The data raises awareness, informs about susceptibility within the community and also informs about phenotypic changes. • This data supports disease model framing in tropical environments. • Data on monthly-annual and geographical trends supports development of public health policies.

## 1 Data

All data in this article were mined from web portals like MAMPU [1], NCBI [2] and NOAA [3]. While year-round weather data (temperature, humidity and wind) were not available for all states, strategic location of Melaka, Pulau Pinang and Sarawak made them ideal to cover the Malaysian Peninsular and Borneo. This data is prepared as monthly average [4] and presented from years 2010–2017 (Table 1). Historical records for hand, foot and mouth disease were retrieved from MAMPU, reorganized into monthly and annual pools before divided into annual age- (Table 2) and case-groups (Table 3). Data comprising of hand, foot and mouth disease associated viruses were extracted from NCBI before separated by collection dates, serotypes and nucleotide codes (Table 4).

**Table 1:** Weather data by monthly average between years 2010 and 2017 for the Malaysian states Pulau Pinang, Melaka and Sarawak.

Month-Year	Pulau Pinang			Melaka			Sarawak		
	Temp. (°C)	Humid. (%)	Wind. (km/h)	Temp. (°C)	Humid. (%)	Wind. (km/h)	Temp. (°C)	Humid. (%)	Wind. (km/h)
Jan-10	28.3	74.2	7.5	27.3	73.3	8.9	26.0	74.9	6.3
Feb-10	29.4	75.8	7.4	28.5	74.3	9.0	26.6	76.2	6.4
Mar-10	29.1	76.4	7.5	28.3	75.2	7.4	26.5	75.8	6.3
Apr-10	29.2	78.2	6.8	28.3	76.6	5.9	27.2	76.3	6.2
May-10	29.5	78.7	6.3	28.6	77.4	5.4	27.6	77.0	6.1
Jun-10	28.2	77.1	6.2	28.0	76.0	5.4	27.3	75.3	5.9
Jul-10	27.8	76.0	6.8	27.4	75.2	5.2	26.4	75.1	6.4
Aug-10	28.3	76.8	6.8	27.3	75.3	5.6	26.6	74.5	6.4
Sep-10	27.8	76.3	6.6	27.3	75.3	5.1	26.5	74.4	6.1
Oct-10	28.2	76.4	5.8	28.0	75.2	5.4	26.6	74.5	7.0
Nov-10	27.2	76.3	6.4	27.2	74.9	5.8	26.3	74.7	6.6
Dec-10	26.5	75.0	6.4	26.4	74.2	5.7	25.8	74.1	6.2
Jan-11	27.1	73.1	7.0	25.8	73.0	7.3	25.6	73.9	6.5
Feb-11	28.1	74.5	7.4	27.4	73.5	7.9	25.9	74.2	6.4
Mar-11	27.1	75.9	6.5	27.3	74.7	6.4	26.3	74.3	6.4
Apr-11	28.1	76.7	6.5	27.8	75.5	6.0	26.6	75.4	6.4
May-11	28.3	77.4	5.8	28.1	76.5	5.1	26.9	75.7	6.2
Jun-11	28.5	76.9	6.3	27.9	76.7	5.4	26.8	75.1	6.1
Jul-11	28.3	76.6	7.0	27.7	76.0	6.0	27.0	74.8	6.1
Aug-11	27.5	76.1	6.9	27.5	75.7	5.9	27.2	75.3	6.4
Sep-11	27.5	76.2	6.4	27.4	75.8	5.5	26.8	75.1	6.6
Oct-11	27.3	76.6	6.2	27.1	75.6	5.5	26.2	75.3	6.1
Nov-11	27.2	76.1	6.6	26.9	75.8	5.6	26.5	75.2	6.9
Dec-11	27.5	74.5	7.2	26.4	74.9	5.8	26.2	75.4	6.7
Jan-12	27.9	74.2	7.0	27.0	74.4	6.2	26.1	74.8	6.9
Feb-12	28.0	76.1	7.4	27.2	75.4	6.2	26.0	74.7	6.2
Mar-12	27.7	76.2	6.3	27.4	75.8	5.4	26.4	75.1	6.2
Apr-12	28.1	77.2	6.5	27.7	76.4	5.6	26.9	75.5	6.6
May-12	28.2	77.2	5.9	28.0	76.6	5.3	27.7	76.0	6.3
Jun-12	28.7	77.4	6.3	28.2	76.5	5.7	27.6	74.8	6.4
Jul-12	27.9	76.0	7.1	27.3	75.9	5.4	26.8	75.0	6.2
Aug-12	28.2	75.4	6.9	27.4	75.9	5.7	27.1	75.1	6.2
Sep-12	27.5	75.0	6.5	27.7	76.1	5.5	27.5	74.9	6.2
Oct-12	27.5	75.6	6.3	27.5	75.7	5.1	26.5	75.1	6.5
Nov-12	27.6	75.3	6.1	26.9	76.0	4.8	26.5	74.9	6.2
Dec-12	27.6	74.4	7.1	26.8	75.6	4.9	26.5	75.0	6.2
Jan-13	28.1	74.8	6.9	27.2	74.7	5.8	26.7	74.9	7.0
Feb-13	27.8	75.1	7.4	26.5	74.8	5.5	26.6	75.1	6.6
Mar-13	29.3	77.9	7.0	28.5	76.6	5.7	27.5	75.5	6.9
Apr-13	28.8	77.7	6.4	28.5	77.4	5.1	27.2	76.0	6.9
May-13	29.3	77.7	6.6	28.3	77.2	4.9	27.2	76.4	6.7
Jun-13	28.8	76.8	6.2	28.8	75.8	5.6	27.7	74.8	6.7
Jul-13	28.3	76.4	5.9	27.6	75.4	5.2	27.0	73.6	7.1
Aug-13	28.1	76.2	7.2	27.7	75.4	5.8	27.2	74.0	7.6
Sep-13	27.8	76.2	6.4	27.3	75.3	5.5	26.7	74.7	7.1
Oct-13	27.3	75.7	6.4	27.5	75.4	6.0	26.7	74.6	7.4
Nov-13	27.8	76.2	6.4	27.3	75.8	5.4	26.2	74.6	6.9
Dec-13	28.1	74.9	7.7	26.7	74.6	7.5	26.1	75.2	7.6
Jan-14	27.9	70.8	8.4	26.4	71.9	9.9	25.4	72.9	7.5
Feb-14	28.6	73.5	8.0	27.9	72.6	10.0	26.5	73.7	6.9
Mar-14	29.4	75.7	8.0	28.1	73.7	8.6	26.7	74.9	6.6
Apr-14	28.7	78.1	6.8	28.2	76.6	5.3	26.9	75.4	6.7
May-14	28.6	78.2	6.5	28.4	77.2	5.4	27.6	76.7	6.4
Jun-14	29.4	79.0	6.8	29.0	77.3	5.6	28.2	76.8	6.2
Jul-14	29.0	77.7	6.2	28.2	76.8	5.2	28.5	75.1	6.8
Aug-14	27.7	76.5	7.3	27.3	76.1	5.7	26.7	74.4	6.4
Sep-14	27.9	76.4	7.0	27.5	75.5	5.4	27.2	73.9	7.3
Oct-14	27.9	76.8	7.0	27.8	75.9	5.4	26.9	74.3	6.5
Nov-14	27.9	76.7	7.1	27.1	75.9	5.4	26.6	74.8	7.0
Dec-14	27.2	74.8	7.0	26.7	75.3	5.9	26.5	74.4	6.4
Jan-15	27.9	72.3	7.6	26.7	72.8	8.5	25.6	73.5	7.2
Feb-15	28.7	72.1	8.2	27.4	72.7	9.3	25.9	73.8	6.6
Mar-15	28.8	75.2	7.4	28.1	75.2	6.9	26.9	74.3	6.7
Apr-15	28.7	77.9	6.5	28.4	77.7	5.3	27.4	76.1	6.6
May-15	28.4	77.4	6.3	28.3	77.3	5.2	27.3	76.0	6.6
Jun-15	28.8	77.3	6.6	28.6	77.8	5.6	28.6	75.7	6.6
Jul-15	27.7	76.0	6.5	28.1	76.7	5.7	27.2	75.2	6.5
Aug-15	27.7	75.7	6.2	28.0	77.3	5.7	27.7	75.7	6.6
Sep-15	26.7	73.7	6.4	28.1	76.7	4.6	27.4	75.7	5.9
Oct-15	26.4	73.8	6.9	27.5	77.0	4.9	26.7	75.9	6.2
Nov-15	26.7	73.5	6.6	27.6	76.9	5.8	26.8	76.1	6.6
Dec-15	27.8	72.5	7.4	28.2	76.7	7.4	27.2	76.6	6.4
Jan-16	28.1	73.3	7.3	28.5	77.0	7.7	27.2	76.8	6.4
Feb-16	28.1	72.0	8.8	27.9	75.0	9.5	26.6	76.3	6.9
Mar-16	29.1	74.9	7.5	29.3	75.4	8.5	27.2	77.0	6.3
Apr-16	29.1	75.5	6.7	29.6	77.6	6.1	27.6	76.8	6.8
May-16	28.0	75.6	6.6	28.9	77.7	5.2	27.5	76.1	6.3
Jun-16	28.5	74.9	6.5	28.2	76.6	5.3	26.9	75.2	6.5
Jul-16	28.5	76.2	3.5	28.0	76.1	3.2	27.6	74.9	3.4
Aug-16	28.4	76.3	6.0	28.6	76.4	4.9	27.7	74.9	7.1
Sep-16	28.0	75.7	6.5	28.3	75.9	5.6	26.9	74.4	7.7
Oct-16	27.5	76.3	5.7	28.3	75.8	6.1	26.8	75.0	7.3
Nov-16	27.5	76.7	6.4	27.3	76.4	5.1	26.7	75.3	6.6
Dec-16	27.5	75.5	6.6	27.3	75.6	5.6	26.8	75.4	6.9
Jan-17	27.3	74.7	6.7	27.0	75.1	5.6	26.7	75.3	6.2
Feb-17	28.0	73.3	7.6	27.1	74.8	6.8	26.3	74.1	6.8
Mar-17	28.0	75.9	6.6	27.4	75.6	5.6	26.9	74.8	6.4
Apr-17	28.2	76.5	6.5	28.0	76.4	5.0	26.6	75.4	6.6
May-17	28.8	77.9	6.9	28.2	76.7	4.9	27.3	76.3	6.5
Jun-17	28.7	76.5	6.6	27.9	75.9	4.7	27.2	75.3	6.2
Jul-17	28.5	76.8	6.7	28.1	75.8	5.2	27.3	74.7	6.6
Aug-17	28.1	76.1	7.2	27.5	75.5	5.0	27.0	74.6	6.3
Sep-17	27.6	76.1	6.5	27.5	75.3	4.8	27.0	75.3	6.3
Oct-17	28.3	75.8	6.6	27.8	75.8	5.1	26.9	74.9	6.8
Nov-17	27.5	76.1	6.4	27.1	75.8	5.2	26.9	75.6	6.4
Dec-17	28.0	73.3	7.5	27.2	74.5	6.5	26.6	75.3	6.9

Note: Weather data are abbreviated as ‘temp.’ to represent air temperature, ‘humid.’ to represent relative humidity and ‘wind.’ to represent wind speed. Weather of Pulau Pinang and Melaka represents Peninsular Malaysia whereas Sarawak represents Borneo.

**Table 2:** Different age groups that contracted hand, foot and mouth disease in every Malaysian state between years 2010 and 2017.

Age group	Johor	Kedah	Kelantan	Melaka	Negeri Sembilan	Pahang	Perak	Perlis	Pulau pinang	Sabah	Sarawak	Selangor	Terengganu	Kuala Lumpur	Labuan
**Child**															
2010	974	880	164	237	327	234	657	89	1458	189	5037	1808	148	966	0
2011	486	274	105	262	189	112	229	31	546	88	2862	1160	72	354	2
2012	2257	1069	1342	768	741	351	1231	216	1543	1569	13212	6244	270	2448	323
2013	1816	737	387	352	660	217	822	207	1193	1548	8315	4998	164	1043	93
2014	2215	966	655	780	1006	233	1620	277	1436	545	9317	8679	393	2300	38
2015	2017	443	228	437	539	170	1056	86	738	1334	8553	4440	169	1274	315
2016	3974	1705	991	2172	1344	816	2695	577	2985	2533	6524	14327	803	3915	246
2017	2256	960	579	1100	895	484	1607	227	1486	1862	7105	7034	278	2276	268
**Adolescense**															
2010	2	3	1	3	1	0	1	0	6	0	8	7	0	8	0
2011	0	0	0	2	0	0	0	0	5	0	11	5	1	2	0
2012	6	2	9	6	0	1	12	0	6	8	36	42	2	7	0
2013	4	1	1	2	0	0	11	0	4	9	39	35	1	5	1
2014	8	1	3	1	0	0	9	0	4	1	39	57	2	9	0
2015	1	1	1	0	0	0	15	0	2	3	44	43	1	9	4
2016	16	3	5	1	8	0	31	0	8	10	22	57	1	19	1
2017	11	1	2	5	3	0	20	0	11	11	34	46	1	12	1
**Adult**															
2010	9	14	2	5	2	0	6	1	29	7	46	38	1	25	0
2011	14	10	7	4	1	3	8	1	21	5	74	42	2	9	0
2012	54	27	48	18	2	3	33	2	30	64	161	280	6	53	7
2013	20	6	12	6	5	0	40	3	8	52	168	284	8	47	5
2014	24	8	10	11	5	0	26	0	10	13	123	383	20	88	0
2015	23	4	4	6	3	1	49	0	18	47	151	272	3	70	7
2016	34	8	10	28	31	6	81	2	26	70	83	682	7	136	3
2017	38	5	6	11	25	1	49	1	55	54	90	366	2	71	5
**Senior adult**															
2010	0	0	0	0	0	0	0	0	0	0	1	0	0	0	0
2011	0	0	0	0	0	0	0	0	1	1	0	1	0	0	0
2012	1	1	0	0	0	0	0	0	0	0	2	5	0	1	0
2013	0	0	1	0	0	0	0	0	0	0	1	0	0	0	0
2014	1	1	0	0	0	0	0	0	0	0	2	2	0	1	0
2015	0	0	0	0	0	0	0	0	0	0	1	4	0	0	1
2016	1	0	1	1	0	0	0	0	0	0	0	8	0	1	0
2017	1	0	0	2	0	0	0	0	1	0	0	1	0	0	0

Note: The age groups are classified as child = 0–14 years, adolescence = 15–19 years, adult = 20–59 years and, senior adult = 60 to >75 years.

**Table 3:** Scale of hand, foot and mouth disease outbreaks in every state between years 2010 and 2017.

Month	Johor	Kedah	Kelantan	Melaka	Negeri Sembilan	Pahang	Perak	Perlis	Pulau Pinang	Sabah	Sarawak	Selangor	Terengganu	Kuala Lumpur	Labuan
**2010**															
Jan	26	28	7	11	9	6	11	6	46	1	450	72	3	24	0
Feb	49	23	12	14	8	4	35	2	50	0	704	41	4	24	0
Mar	61	37	8	11	7	8	44	6	100	1	1050	151	11	61	0
Apr	51	37	9	18	10	22	59	4	64	8	543	147	24	37	0
May	95	74	19	42	87	45	121	5	178	47	381	306	24	106	0
Jun	107	118	24	26	59	36	100	4	252	20	348	306	19	129	0
Jul	112	192	16	35	66	51	134	11	216	22	349	214	22	130	0
Aug	146	221	34	35	33	36	89	30	158	22	425	237	29	243	0
Sep	52	54	10	17	14	8	24	5	159	10	162	93	4	70	0
Oct	122	64	12	22	15	6	20	12	137	36	274	144	7	121	0
Nov	121	30	9	10	17	3	15	1	78	21	174	73	2	33	0
Dec	43	19	7	4	5	9	12	4	55	8	232	69	0	21	0
**2011**															
Jan	12	9	2	3	8	3	9	2	35	1	6	24	2	5	0
Feb	14	4	5	4	5	9	13	1	38	0	34	27	4	30	0
Mar	17	9	4	14	13	7	14	1	34	1	233	75	2	3	2
Apr	41	3	5	6	18	6	11	1	25	1	128	83	7	10	0
May	26	11	0	25	9	13	15	2	51	0	109	110	2	4	0
Jun	40	15	2	21	13	7	39	3	24	1	125	50	1	31	0
Jul	114	16	3	22	18	2	5	2	37	3	126	78	3	28	0
Aug	51	13	2	52	15	6	19	3	30	2	277	72	3	7	0
Sep	28	21	5	13	13	2	12	1	20	3	259	43	12	0	0
Oct	59	73	8	33	28	20	30	5	47	11	404	155	25	99	0
Nov	44	60	23	22	31	14	46	11	95	7	483	256	6	86	0
Dec	54	50	53	53	19	26	24	0	137	64	763	235	8	62	0
**2012**															
Jan	42	27	31	13	23	5	30	10	83	9	256	102	9	55	1
Feb	55	53	118	63	46	19	33	16	89	113	818	321	39	140	0
Mar	298	138	466	129	184	52	146	31	126	265	2698	1013	94	179	180
Apr	487	160	361	104	160	109	294	17	193	260	1263	1259	40	486	135
May	539	153	174	210	95	44	265	36	250	309	1060	1343	33	533	8
Jun	379	172	136	107	73	68	160	41	235	159	1837	950	15	583	0
Jul	172	117	64	66	54	26	79	21	179	94	1317	460	16	145	0
Aug	97	105	17	44	35	16	75	17	98	65	1080	296	6	72	0
Sep	86	76	7	23	27	7	68	3	127	112	1301	259	12	94	0
Oct	78	37	7	7	21	3	46	19	67	114	764	216	5	78	3
Nov	50	24	9	14	13	2	28	3	60	74	613	184	7	3	2
Dec	35	37	9	12	12	4	52	4	72	67	404	168	2	141	1
**2013**															
Jan	51	28	21	18	18	4	36	6	24	32	683	184	7	91	3
Feb	77	37	24	41	30	12	45	5	32	76	1273	309	10	70	7
Mar	278	63	57	60	71	36	144	13	72	198	2105	726	30	126	10
Apr	178	66	38	41	63	12	107	14	76	304	1136	584	18	128	4
May	238	53	43	37	61	36	88	21	119	340	795	563	14	103	14
Jun	224	97	52	42	81	29	111	39	213	319	606	562	19	114	27
Jul	138	91	32	16	74	24	83	25	185	166	289	320	11	57	25
Aug	88	74	33	13	62	10	66	21	115	56	217	349	13	70	2
Sep	145	64	28	31	64	15	63	15	117	35	271	493	9	84	2
Oct	178	40	25	22	54	12	46	15	78	27	328	443	16	79	2
Nov	158	71	29	18	65	23	60	20	89	40	521	500	15	99	2
Dec	87	60	19	21	22	4	24	16	85	16	299	284	11	74	1
**2014**															
Jan	47	51	28	47	23	18	80	17	75	16	368	298	26	89	1
Feb	68	87	28	83	37	32	114	29	32	36	677	469	44	80	0
Mar	295	101	80	89	88	20	266	62	101	116	1331	753	83	164	7
Apr	278	95	50	65	113	14	202	19	157	48	1208	781	32	193	1
May	271	104	46	91	125	25	226	28	253	54	1535	1000	11	353	0
Jun	132	73	23	43	43	20	75	28	132	35	858	400	15	143	1
Jul	88	41	28	33	57	12	64	13	73	46	669	197	15	60	1
Aug	91	45	51	26	59	18	65	4	49	48	665	393	26	106	3
Sep	130	64	55	46	113	16	103	10	54	31	628	659	34	174	0
Oct	278	76	84	64	97	19	104	18	109	37	455	964	42	259	7
Nov	317	133	121	122	161	15	180	23	209	55	599	1820	50	436	10
Dec	253	106	74	83	95	24	176	26	206	37	488	1387	37	341	7
**2015**															
Jan	278	86	68	54	136	35	188	15	173	119	1348	1197	37	284	5
Feb	327	72	48	57	107	40	267	17	92	189	2264	783	31	172	25
Mar	254	42	29	80	83	26	158	10	73	229	2329	632	19	164	74
Apr	341	29	22	52	52	11	141	8	104	228	1348	581	15	183	63
May	277	58	14	32	46	4	111	12	74	196	849	470	17	138	62
Jun	105	18	8	24	27	8	58	10	41	100	312	192	15	65	12
Jul	68	22	4	10	19	5	22	2	15	55	100	138	3	37	7
Aug	129	26	6	37	16	8	43	6	40	44	74	207	1	63	15
Sep	82	21	10	33	14	2	28	1	29	39	38	132	4	44	23
Oct	91	23	7	30	13	14	42	3	62	58	36	142	6	71	24
Nov	51	24	8	23	21	10	19	2	42	90	35	133	15	63	14
Dec	38	27	9	11	8	8	43	0	13	37	16	152	10	69	3
**2016**															
Jan	93	21	12	25	32	11	48	9	30	97	70	224	8	88	6
Feb	101	26	18	39	53	14	104	9	22	216	52	330	13	102	10
Mar	205	23	23	62	67	32	91	10	55	319	70	447	16	127	32
Apr	308	49	35	161	112	82	175	24	115	320	240	823	34	307	66
May	436	76	35	290	133	134	347	74	301	302	393	1750	72	486	27
Jun	440	160	58	224	270	113	271	62	348	140	707	1764	67	445	14
Jul	508	193	120	274	164	93	278	96	370	130	1235	1544	77	422	14
Aug	757	377	152	403	207	74	365	82	413	104	1313	2229	171	535	20
Sep	378	239	151	205	93	24	286	57	307	65	892	1352	120	329	9
Oct	388	255	175	201	125	96	326	59	349	169	623	1689	101	439	21
Nov	191	156	131	177	52	69	215	55	315	292	561	1383	74	351	8
Dec	220	141	97	141	75	80	301	42	394	459	473	1539	58	440	23
**2017**															
Jan	255	158	101	94	94	67	252	26	263	508	823	1061	48	276	40
Feb	295	137	89	142	115	71	302	29	210	508	1363	1143	42	285	33
Mar	325	83	55	167	105	75	252	24	139	338	1831	951	30	259	36
Apr	307	108	49	145	106	51	202	21	146	167	1310	860	28	330	64
May	211	63	30	118	44	31	112	9	162	87	525	566	13	175	25
Jun	109	58	19	94	69	16	101	13	94	50	388	420	9	123	9
Jul	147	70	32	103	45	22	73	17	103	38	393	480	19	125	11
Aug	220	84	35	69	49	37	82	29	101	27	191	469	20	159	11
Sep	142	54	43	61	95	35	96	18	90	36	121	400	15	139	6
Oct	100	48	31	43	67	28	63	7	115	27	83	341	13	160	4
Nov	94	49	53	34	75	25	75	11	56	79	113	319	16	169	3
Dec	101	54	50	48	59	27	66	24	74	62	88	437	28	159	32

**Table 4:** Types of viruses associated with hand, foot and mouth disease in Malaysia.

Johor	Kedah	Kelantan	Melaka	Pahang	Pulau pinang	Sarawak	Selangor	Terengganu	Kuala Lumpur
**2010**									
KC894870.1 (Apr) [4] KC894871.1 KC894874.1 (May) [4] KC894880.1 (Aug) [4]	KC894876.1 (Jul) [4]	KC894886.1 (Apr 2012) [4]	KC894877.1 KC894878.1 KC894879.1 (Aug) [4]	*	KC894873.1 (May) [4]	*	KF734978.1 KP890662.1 KP890663.1 (Oct) [13] KJ675505.1 KJ675507.1 KJ675506.1 (Nov) [13]	KC894872.1 (May) [4] KC894875.1 (Jun) [4]	KC894881.1 (Sep) [4] JQ356548.1 JQ356552.1 JQ356554.1 (Oct) [13] JQ356547.1 JQ356551.1 JQ356560.1 ***JQ356571.1*** (Nov) **[*11***, 13] JQ356564.1 (Dec) [13]
**2011**									
*	*	*	*	KP205031.1 (Mar) [9] KP205033.1 (May) [9] KP205032.1 (Aug) [9] KC894867.1 (Nov) [4]	*	KC894868.1 KC894869.1 (Sep) [4]	KP890664.1 (May) [13]	KC894865.1 (Sep) [4] KC894866.1 (Oct) [4]	JQ356556.1 JQ356557.1 (Jan) [13] JQ356558.1 (Feb) [13] ***KP205031.1*** JQ356563.1 (Mar) [***9***, 13] Q356555.1 (Apr) [13] ***KP205033.1*** JQ356559.1 (May) [***9***,13] KP205032.1 (Aug) [13] JQ356568.1 (Nov) [9]
**2012**									
KC894882.1 KC894883.1 KC894887.1 KC894888.1 KC894891.1 KC894892.1 (Apr) [4] KC894889.1 KC894890.1 KC894893.1 KC894894.1 KC894896.1 KC894897.1 KC894898.1 (May) [4] KC894899.1 KC894900.1 (June) [4] KC894902.1 (Aug) [4]	KC894884.1 (Apr) [4] KC894895.1 (Aug) [4]	*	*	KC894885.1 (Apr) [4] KC894903.1 (Jun) [4] KP205034.1 KP205035.1 (Oct) [3]	*	*	KC894901.1 (Jul) [4] KJ728862.1 KJ728863.1 KJ728864.1 ***KJ728868.1*** (Dec) [***6***,11]	*	*KJ815033.1* *KT908016.1* ***KT908015.1*** ***KT908038.1*** KT908004.1 KT908005.1 KT908006.1 KT908026.1 KT908027.1 KT908028.1 KT908029.1 (May) [*1*,***2***,4] ***KJ815034.1*** ***KT908017.1*** KT908007.1 KT908031.1 KT908032.1 (Jun) [***1***,4] ***KJ815035.1*** ***KT908018.1*** KT908008.1 KT908009.1 KT908010.1 KT908011.1 KT908033.1 KT908034.1 (Jul) [***1***,4] ***KP205034.1*** ***KP205035.1*** KT908012.1 KT908013.1 KT908035.1 KT908036.1 (Aug) [*1*,***3***,4] KP205034.1 KP205035.1 (Nov) [6] KJ815037.1 KT908019.1 (Dec) [1]
**2013**									
*	*	*	*	*KR068478.1* *KR068480.1* ***KP205038.1*** ***KP205039.1*** KP205036.1 KP205037.1 (May) [*4*,***8***,9] KR068477.1 KR068479.1 (Jul) [4]	*	*	*KF772887.1* *KF772888.1* *KF772889.1* *KF772904.1* *KF772905.1* *KF772906.1* **KJ728867.1** **KJ728866.1** **KJ728865.1** ***KJ728858.1*** ***KJ728859.1*** KJ728861.1 (Jan)[*1*,**11**,***12***,13] *KF772907.1* *KF772890.1* ***KF772894.1*** ***KF772899.1*** KF772901.1 KF772896.1 (Mar) [*1*,***2***,4] *KF772908.1* *KF772891.1* *KF772909.1* *KF772892.1* ***KF772902.1*** ***KF772897.1*** ***KF772903.1*** ***KF772898.1*** KJ728860.1 (Apr) [1], [4], [13] KF772900.1 KF772895.1 (Jun) [2] KF772900.1 KF772887.1 KF772910.1 KF772893.1 (Aug) [1]	*	***KJ815038.1*** ***KJ815039.1*** ***KT908020.1*** KT908014.1 KT908037.1 (Apr) [***1***,4] *KR068478.1* *KR068480.1* ***KP205039.1*** ***KP205038.1*** KP205036.1 KP205037.1 (May) [*4*,***8***,9] KR068477.1 KR068479.1 (Jul) [5] KJ815040.1 KJ815041.1 KJ815042.1 KJ815043.1 KJ815044.1 KT908021.1 KT908022.1 KT908023.1 KT908024.1 KT908025.1 (Sep) [1]
**2014**									
*	*	*	*	***KP204987.1*** KP205006.1 (Nov) [***7***,10]	*	*	*	*	*

Note: Data in this table were extracted from published nucleotide sequences in National Centre for Biotechnology Information. Picornaviridae members are defined using 1 = Coxsackie A6, 2 = Coxsackie A16, 3 = Coxsackie B3, 4 = Enterovirus A71, 5 = Enterovirus B5, 6 = Enterovirus D68, 7 = Echovirus 1, 8 = Echovirus 6, 9 = Echovirus 7, 10 = Echovirus 11, 11 = Rhinovirus A, 12 = Rhinovirus B and, 13 = Rhinovirus C. Keys in round (brackets) = the period of virus isolation whereas box [brackets] = virus serotypes. Information on HFMD causing viruses are not available for the states Labuan, Perak, Perlis and Sabah.

## 2 Experimental design, materials, and methods

Historical weather (temperature, humidity and wind) data were retrieved from National Weather Service (NOAA) historical records that range from years 2010–2017. The sorting and arrangement of historical weather data followed procedures of Nelson et al. [5], [6]. Data mining from web portals like MAMPU and NCBI adopted a two-way inclusion keyword system [7], [8], [9], [10]. In this case, abbreviated keys like ‘CVA’, ‘CVB’, ‘EO7’, ‘HFMD’, ‘EV71’, ‘EVA’, ‘EVB’, ‘EVC’, ‘RHA’, ‘RHB’ and ‘RHC’ and-or virus names like ‘Coxsackivirus’, ‘Echovirus’, ‘Enterovirus’, ‘Rhinovirus’ were added to the primary keyword ‘Malaysia’. After first data extraction, the abbreviated keys and-or virus names were added with ‘Sabah’, ‘Sarawak’, ‘Labuan’, ‘Perak’, ‘Pahang’, ‘Kelantan’, ‘Johor’, ‘Negeri Sembilan’, ‘Perlis’, ‘Pulau Pinang’, ‘Penang’, ‘Kuala Lumpur’, ‘Selangor’ and ‘Terengganu’ as word strings. Notably, all nucleotide ascensions were sorted by period of publication, from 01 January 2010 to 31 December 2018. Information related to molecular ecology were first divided by ascension numbers and location sources [11], [12]. The crude data were then filtered and sorted first [13], [14], [15] before adapted with hierarchy-identification code system [16], [17] to ease data comprehension and also extraction.
