# Supplementary material for: Compendium of hand, foot and mouth disease data in Malaysia from years 2010–2017
Source: Data Brief. 2019 Mar 20;24:103868. doi: 10.1016/j.dib.2019.103868 (PMC6441719; doi:10.1016/j.dib.2019.103868)
Supplement: Multimedia component 1 [file mmc1.doc]

Conflict of Interest and Authorship Conformation Form

Please check the following as appropriate:

( / ) All authors have participated in (a) conception and design, or analysis and interpretation of the data; (b) drafting the article or revising it critically for important intellectual content; and (c) approval of the final version.

( / ) This manuscript has not been submitted to, nor is under review at, another journal or other publishing venue.

( / ) The authors have no affiliation with any organization with a direct or indirect financial interest in the subject matter discussed in the manuscript

Author’s name Affiliation

1) Bryan Raveen Nelson Institute of Tropical Biodiversity and Sustainable Development, Universiti Malaysia Terengganu

________________________________________________________________________

2) Edinur Hisham Atan School of Health Sciences, Universiti Sains Malaysia

3) Mohd Tajuddin Abdullah School of Marine and Environment Sciences Universiti Malaysia Terengganu

Institute of Tropical Biodiversity and Sustainable Development, Universiti Malaysia Terengganu
